# Compromised Mitochondrial Fatty Acid Synthesis in Transgenic Mice Results in Defective Protein Lipoylation and Energy Disequilibrium

**DOI:** 10.1371/journal.pone.0047196

**Published:** 2012-10-15

**Authors:** Stuart Smith, Andrzej Witkowski, Ayesha Moghul, Yuko Yoshinaga, Michael Nefedov, Pieter de Jong, Dejiang Feng, Loren Fong, Yiping Tu, Yan Hu, Stephen G. Young, Thomas Pham, Carling Cheung, Shana M. Katzman, Martin D. Brand, Casey L. Quinlan, Marcel Fens, Frans Kuypers, Stephanie Misquitta, Stephen M. Griffey, Son Tran, Afshin Gharib, Jens Knudsen, Hans Kristian Hannibal-Bach, Grace Wang, Sandra Larkin, Jennifer Thweatt, Saloni Pasta

**Affiliations:** 1 Children's Hospital Oakland Research Institute, Oakland, California, United States of America; 2 Department of Medicine, University of California Los Angeles, Los Angeles, California, United States of America; 3 Buck Institute for Research on Aging, Novato, California, United States of America; 4 School of Veterinary Medicine, University of California Davis, Davis, California, United States of America; 5 Dominican University of California, San Rafael, California, United States of America; 6 Department of Biochemistry and Molecular Biology, Odense University, Odense, Denmark; Max Delbrueck Center for Molecular Medicine, Germany

## Abstract

A mouse model with compromised mitochondrial fatty acid synthesis has been engineered in order to assess the role of this pathway in mitochondrial function and overall health. Reduction in the expression of mitochondrial malonyl CoA-acyl carrier protein transacylase, a key enzyme in the pathway encoded by the nuclear *Mcat* gene, was achieved to varying extents in all examined tissues employing tamoxifen-inducible *Cre*-*lox* technology. Although affected mice consumed more food than control animals, they failed to gain weight, were less physically active, suffered from loss of white adipose tissue, reduced muscle strength, kyphosis, alopecia, hypothermia and shortened lifespan. The Mcat-deficient phenotype is attributed primarily to reduced synthesis, in several tissues, of the octanoyl precursors required for the posttranslational lipoylation of pyruvate and α-ketoglutarate dehydrogenase complexes, resulting in diminished capacity of the citric acid cycle and disruption of energy metabolism. The presence of an alternative lipoylation pathway that utilizes exogenous free lipoate appears restricted to liver and alone is insufficient for preservation of normal energy metabolism. Thus, de novo synthesis of precursors for the protein lipoylation pathway plays a vital role in maintenance of mitochondrial function and overall vigor.

## Introduction

According to the United Mitochondrial Disease Foundation, each year in the United States, as many as 4,000 children are born who will develop a mitochondrial disease by age 10. On the other hand, many patients suffer symptoms for years before they are accurately diagnosed and some mitochondrial defects manifest only in adults as diseases of aging. Because mitochondria perform many different functions in addition to ATP synthesis, there likely are hundreds of diseases that can result from mitochondrial defects, each disorder producing a spectrum of abnormalities that can be confusing to diagnose [1], [2]. One of the difficulties in unraveling the etiology of mitochondrial disorders has been that the roles of many of the ∼1500 mitochondrial proteins are poorly understood and mutations in different genes, encoded in either the mitochondrial or nuclear genomes, can lead to similar diseases. Only recently has it become clear that human mitochondria contain their own unique system for synthesizing fatty acids de novo that is encoded by nuclear genes [3], [4], [5], [6], [7], [8]. These genes encode a suite of discrete individual proteins, termed type II fatty acid synthase, that is quite distinct from the megasynthase complex responsible for de novo fatty acid synthesis in the cytosolic compartment, the type I fatty acid synthase, in which the individual enzymes are covalently linked. In vitro studies [9], [10] have revealed that the major product of the mitochondrial pathway is an octanoyl moiety that can be utilized as a precursor for the formation of lipoyl moieties that are utilized for the posttranslation modification of key mitochondrial proteins that constitute the lipoamide subproteome: the E2 subunits of the pyruvate (PDC), α-ketoglutarate (KDC) and branched-chain α-ketoacid (BCDC) dehydrogenase complexes and the H-protein component of the glycine cleavage system (GCS). However, posttranslational modification of mitochondrial proteins reportedly also can be expedited using lipoyl moieties derived from exogenous free lipoic acid [11], [12], so the relative physiological importance of the two lipoylation pathways is unclear. The objective of this study was to resolve this issue by developing a mouse model in which functionality of the mitochondrial pathway for the de novo synthesis of fatty acyl and lipoyl moieties was compromised. The target for knockout was the nuclear gene *Mcat* that encodes the mitochondrial malonyl CoA-acyl carrier protein (ACP) transacylase. Mcat catalyzes the transfer of malonyl moieties from CoA to the phosphopantetheinyl moiety of the acyl carrier protein (ACP) that is responsible for shuttling reaction intermediates through the catalytic centers of the enzymes participating in the pathway [3]. The structure and mechanism of action of Mcat has been elucidated [13].

## Methods

### Engineering and genotyping of the knockout mouse models

A tamoxifen-inducible knockout of the mitochondrial *Mcat* gene (GenBank accession number BC099494) was engineered using *Cre*-*lox* technology as follows. The mitochondrial *Mcat* gene was initially identified by screening a 129-Ola BAC library and used to construct a promoterless gene-trap vector [14], essentially as described earlier [15], [16], [17]. A modified *Mcat* gene was engineered with Lox sites flanking exon 2 to allow eventual excision of exon 2 by a *Cre* recombinase, resulting in a frame shift in exon 3 and the production of a severely truncated protein (Fig. S1). The gene-trap cassette was inserted by homologous recombination so as to generate an in-frame fusion protein that facilitated expression of the encoded selectable marker in mouse embryonic stem cells. The cells were electroporated with linearized DNA, 24 selected colonies were picked and RNA isolated and analyzed by 5′-RACE. Ten targeted clones were identified and authenticity confirmed by sequencing and by PCR. Chosen clones were treated with *Flp* recombinase in vitro, to excise the *Frt*-flanked *ß-geo* selection cassette, then were injected into C57BL/6J blastocysts and the resulting male chimeric mice were outcrossed with female C57BL/6J mice. Germline transmission was found in 66% of the brown offspring. These mice were intercrossed to generate mice homozygous for the floxed *Mcat* allele (HF mice) then crossed with the inducible-*Cre* strain B6.Cg-Tg(cre/Esr1)5Amc/J, obtained from Jackson Labs.

### Genotyping

Tissues were digested with proteinase K and DNA was extracted with phenol:chloro-form:isoamyl alcohol using Phase Lock Gel™ and purified by ethanol precipitation. *Mcat* and *Cre* recombinase genes were characterized using the PCR (Fig. S1).

### Animal husbandry and tamoxifen treatment

Mice were housed communally in Thoren Maxi-Miser ventilated cages maintained at 72±2°F with a 12 h dark, 12 h light cycle and provided water and Teklad Irradiated Diet 2918 ad libitum. At 4–6 weeks of age, mice were given five daily intraperitoneal injections of tamoxifen in corn oil, 0.1 mg per g body weight. In later experiments, in an attempt to minimize stress on the animals, tamoxifen was administered orally. These mice were first fed a reconstituted soy-free diet (Teklad 2020X) for 1 week then transferred to the same diet supplemented with tamoxifen (0.5 mg per g) for 1 month, after which they were returned to the standard diet. Both KO and HF mice typically exhibited transient weight loss during the period of tamoxifen treatment, presumably because of the anorexic properties of this compound.

### Ethics statement

All procedures were approved by the Children's Hospital Oakland Research Institute Animal Care and Use Committee (Protocol #171) and complied with the U.S. Public Health Service Policy on Humane Care and Use of Laboratory Animals. The Children's Hospital Oakland Research Institute Animal Research Facility is certified by the Association for the Assessment and Accreditation of Laboratory Animal Care.

### Food consumption

Food consumption was measured by weighing the amount of food remaining in the food compartment at intervals of 2–3 days and was normalized for the average body weight of animals in the cage.

### Open field analysis

Open field locomotion [18] was assessed by placing the mouse in the center of a wooden box 3ft×3ft marked in 6 inch squares. Posture, gait and behavior were observed and activity level was quantitated by recording the number of squares visited in a 3 min observation period. Each mouse was tested twice within a 2-week period and the results averaged.

### Rotarod performance

An Economex Rota-Rod (Columbus Instruments) was used in the acceleration mode, beginning at 4 rpm and accelerating at 0.1 rpm/sec until the mouse fell from the rotating rod. Each mouse was tested three times at 30 min intervals and the aggregate time on the rotarod recorded. In the endurance mode, rotation was fixed at 5 rpm and, in the event of a fall, the mouse was replaced on the rod.

### Grip strength

The peak force a mouse exerted by the front and rear limbs was measured using a grip strength meter from Columbus Instruments [19].

### Necropsy, histology and blood chemistry

Full necropsies, with histological examination on all major tissues, and plasma chemistry panel analysis were performed by the Comparative Pathology Laboratory at the University of California Davis.

### Muscle and liver glycogen and lactate

Muscle and liver glycogen content was measured using a kit from Biovision, Mountain View, CA. Muscle lactate levels were measured at the Scripps Center for Metabolomics and Mass Spectrometry.

### Hematology

CBC analysis was performed using a Siemens Advia 120 system. Red blood cell turnover rate was assessed using flow cytometry to monitor the disappearance of biotin-labeled red blood cells from the circulation [20]. Injection of mice via the tail vein with EZ-Link sulfo-*N*-hydroxysuccinimide-biotin (Thermo Scientific) resulted in labeling of the entire red blood cell population.

### Fecal analysis

Feces from a 24-h collection were lyophilized and pulverized. Portions of 200 mg were extracted and assayed for protein content, using the BCA reagent (Pierce), essentially as described earlier [21]. Lipids were extracted from 300 mg portions of dried feces with 4 mL chloroform-methanol and quantitated gravimetrically, essentially as described earlier [22]. Hemoglobin ß was assayed in protein extracts by Western analysis using anti-mouse hemoglobin ß antibody (Santa Cruz Biotechnology).

### Isolation of Mitochondria

Mitochondria from all tissues, except spleen and bone marrow, were purified on a five-layer discontinuous iodixanol gradient and used for both Western analysis and enzyme activity assays. The purity of mitochondrial fractions prepared in this way was confirmed by Western analysis using antibodies against a panel of marker proteins [8]. Mitochondria from mouse spleens were purified by differential centrifugation [9], [10].

### Western analysis

Electrophoresis and Western blotting was performed as described previously [8]. Purified mitochondria were used for all tissues except bone marrow, for which whole-cell extracts were used. Sample loading was normalized based on protein content, usually 5 to 10 µg mitochondrial protein/lane. ß-ketoacyl synthase (Oxsm) and prohibitin (Phb) immunostaining served as additional visual loading controls. Each sample was analyzed at least twice, and only unsaturated images that contained a limited numbers of black pixels were used for quantification.

### Enzyme activities

Activities of PDC and KDC were monitored at 340 nm, 30°C, in the presence of 0.1% Triton X-100, as described previously [23] except that components of INT-coupling system and D,L-carnitine were omitted from the assay.

### Overall activity of the mitochondrial fatty acid biosynthetic pathway

The formation of ^14^C-octanoyl-ACP, the major product, from [2-^14^C] malonate was assayed as described earlier [8].

### Respiration of isolated mitochondria

Mitochondria (0.5 mg of protein.mL^−1^), isolated from hind limb skeletal muscle by differential centrifugation [24], were incubated at 37°C in air-saturated medium (120 mM KCl, 5 mM Hepes, 1 mM EGTA, 5 mM KH_2_PO_4_, pH 7.2, supplemented with 0.3% defatted bovine serum albumin), and respiration monitored with an oxygen electrode. Succinate (5 mM) and rotenone (2 µM) were used to assay respiration through complex II. α-Ketoglutarate (7.5 mM) was used to assay respiration through KDC. Pyruvate (100 µM) and malate (5 mM) were used to assay respiration through PDC. State 3 respiration was induced in the presence of substrate by 500 µM ADP, state 4 by the addition of 1 µg.mL^−1^ oligomycin, and uncoupled respiration was measured in the presence of carbonyl cyanide p-(tri-fluromethoxy)phenyl-hydrazone.

### Cyanide titrations in isolated mitochondria

Oxygen consumption rates were measured in skeletal muscle mitochondria isolated from C57BL/6J mice using ascorbate (1.2 mM) as substrate in the presence of N,N,N′,N′-tetramethyl-p-phenylenediamine (0.1 mM) to catalyze electron donation to cytochrome *c* and complex IV. To eliminate the contribution of complexes I, III and V, we added antimycin A (20 µM), rotenone (2 µM) and oligomycin (2 mg.mL^−1^). 4-trifluoromethoxyphenylhydrazone (1 µM) was added to obtain the maximum oxygen consumption rate. A control oxygen consumption rate was obtained then the concentration of cyanide needed to inhibit complex IV was determined, to simulate the degree of cytochrome *a* depletion in the KO (40%).

### Measurements of cytochrome content

Cytochrome content was determined from difference spectra (dithionite-reduced minus air-oxidized) on a DW-2 Olis dual-beam spectrophotometer. Mitochondria (1 mg.mL^−1^) from HF and KO animals were lysed in CP-1 [25] medium plus 0.5% (w/v) sodium deoxycholate. Concentrations of cytochromes *c*, *c_1_*, *b*, and *a* were calculated by solving simultaneous equations as described earlier [26], [27]. Wavelength pairs and extinction coefficients were: cytochrome *c* 550–535 nm, 25.1 mM^−1^.cm^−1^; cytochrome *c_1_* 554–540 nm, 24.1 mM^−1^.cm^−1^; cytochrome *b* 563–577 nm, 23.2 mM^−1^.cm^−1^; cytochrome *a* 605–630 nm, 13.1 mM^−1^.cm^−1^.

### Statistics

Unless otherwise stated, values are reported as means with standard deviations. Significance was assessed using two-tailed independent T-tests.

## Results and Discussion

### Engineering of knockout mice

**Figure 1:**
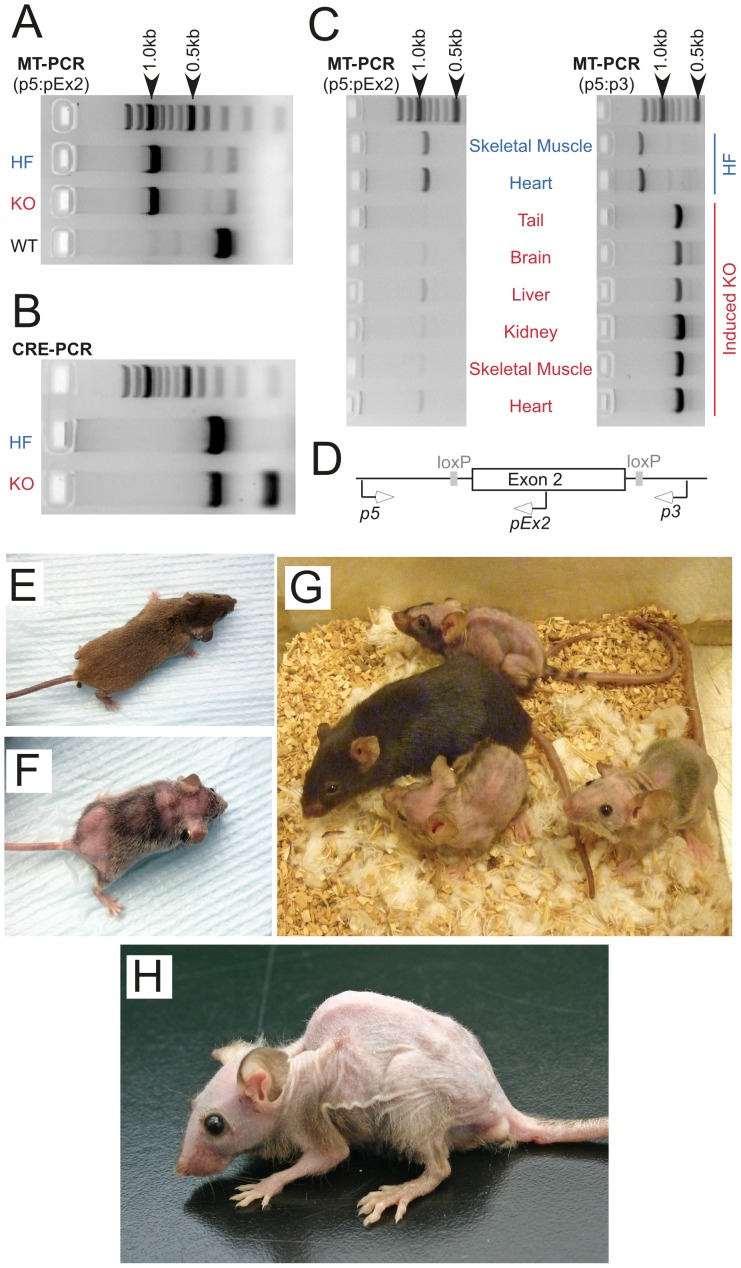
Genotype and phenotype of the *Mcat* knockout mouse. A–D: genotyping by PCR. Mice were screened by PCR analysis of tail DNA, using the primer pair p5:pEx2, which generates 900 and 300 bp products from the floxed and wild-type alleles, respectively (panels A and D), and those carrying the floxed *Mcat* allele were bred to homozygosity (HF). HF mice were mated with *Cre* mice and bred to homozygosity for the HF *Mcat* allele and hemizygosity for the *Cre* gene, to give the KO genotype. The *Cre* gene was detected by the unique 100 bp product amplified in the *Cre*-PCR reaction, in addition to the 300 bp internal positive control (panel B). After treatment with tamoxifen, deletion of exon 2 was detected in various tissues from the KO mice, as judged by two independent PCR reactions (panels C and D). Primer pair p5:pEx2 detects the HF allele (900 bp product) and primer pair p5:p3 generates an 1800 bp product from the HF allele and an 800 bp product from the exon-2-deleted KO allele. Similar results, confirming exon 2 deletion, were observed in skin, bone marrow and lung from KO mice. E–O: phenotyping. E–H, mouse photographs. E and F, Female HF control and KO, respectively, 4 months after tamoxifen treatment. G: Three KO and one HF control female mice 9 months after tamoxifen treatment. H: Male KO mouse 9 months after tamoxifen treatment.

Mice homozygous for a modified mitochondrial malonyl-CoA:ACP acyl transferase gene (*Mcat*) with *lox* sites flanking exon 2 (HF mice) were engineered and crossed with mice of the *Cre* strain B6.Cg-Tg(cre/Esr1)5Amc/J, which carries a single copy of a tamoxifen-inducible, *Cre*-mediated recombination system driven by the chicken ß-actin promoter/CMV immediate-early enhancer that facilitates expression in most tissues. Since mice homozygous for this *Cre* recombinase gene are not viable, progeny testing positive for both the *Cre* recombinase and floxed *Mcat* genes were then mated with HF mice. Progeny homozygous for the floxed *Mcat* allele that also carried a single copy of the *Cre* recombinase gene were identified as knockout (KO) mice. These mice, when mated with HF mice, produced litters that contained equal proportions of HF and KO offspring; the HF littermates were used as control animals for most experiments. Initial genotyping of the transgenic mice was accomplished by analysis of tail DNA using the PCR (Fig. 1 A & B). KO mice and HF control mice were treated with tamoxifen, either via intraperitoneal injection or by inclusion in the diet. The phenotypes induced in KO mice by the two routes were indistinguishable. Tamoxifen promoted excision of exon 2 from the *Mcat* alleles, with variable efficiency, in all tissues tested from KO mice but not in those from HF mice (Fig. 1 panels C and D).

### Appearance and activity of knockout mice

**Figure 2:**
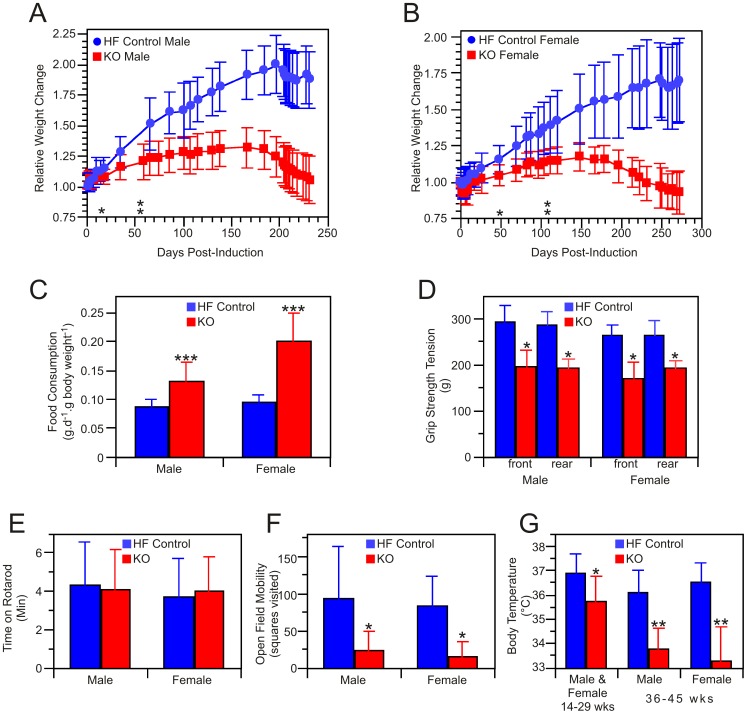
Characteristics of the *Mcat* knockout mouse. A and B: mean relative body weights of 13 KO and 13 HF control male mice, initial weights 21.6±2.5 g and 22.9±2.5, respectively (A) and 10 KO and 10 HF control female mice, initial weights 20.8±3.2 g and 19.0±3.2 g, respectively (B). Asterisks indicate the earliest time points at which statistically significant differences in weight were observed. C: Mean food consumption monitored for a 2-week period 7 months after tamoxifen treatment (11–13 measurements each group). D: mean grip strength of male, 5 KO and 7 HF control, and female, 6 KO and 5 HF control mice. E: Mean residence times on the accelerating rotarod, measured 7 months post-induction, were not significantly different between KO (14 male, 18 female) and HF controls (10 male, 25 female). F: Physical activity in the Open Field Test, assessed 7–8 months after tamoxifen treatment, in KO (12 male, 7 female) and HF controls (10 male, 9 female). G: Rectal body temperatures monitored in the period 14–29 weeks after tamoxifen treatment (9 KO males and 7 KO females combined, 3 HF males and 8 HF females combined; 7 measurements for each mouse) and in the period 36–45 weeks post-treatment (sexes shown separately, 2–3 measurements each mouse). Statistical significance in the T-test, where observed, is indicated by *p>0.05, **p<0.005 or ***p<0.0005.

About 3–4 months after treatment with tamoxifen, the coats of KO mice lost their normal luster and hair loss became noticeable (Fig. 1 panels E and F). This condition became progressively worse, eventually resulting in severe baldness (Fig. 1, panel G). Skin of the KO mice was noticeably dryer in appearance, compared to HF controls, and some of the severely affected mice developed puritus that led to self-inflicted scratch wounds, necessitating euthanasia (according to institutional guidelines). The alopecia was universally accompanied by the development of pronounced kyphosis (Fig. 1, panel H). Both male and female KO mice treated with tamoxifen failed to gain weight as well as their littermate HF controls and after 6 months the KO mice began to lose weight (Fig. 2 panels A and B). The mean survival time for KO mice not euthanized for experimentation was 293±70 days post-induction (n = 13). Weight loss was not observed in HF control mice, even up to one year after treatment with tamoxifen and is not usually observed in normal mice until after the age of 18 months [28]. The failure to thrive could not be attributed to inadequate food intake for, surprisingly, the KO mice ate significantly more food than the HF controls, when normalized for body weight (Fig. 2 panel C). Neither could the condition be ascribed to a defect in digestion, since the protein and lipid content of the feces was not significantly different in the two groups (Table 1). Although grip strength of the KO mice was significantly lower than that of HF controls (Fig. 2, panel D), the KO mice performed as well as the HF controls on the accelerating rotarod, which assesses motor coordination and balance for a short interval, typically 1–2 minutes for each of three tests (Fig. 2, panel E). However, when the rotarod was maintained at constant 5 rpm, in a procedure designed to assess endurance, the KO mice fared less well (these experiments were performed only with female mice). HF control mice were able to complete 3 hours on the device without difficulty, averaging only one fall every 144 min, and remained lively at the end of the experiment. In contrast, KO mice quickly became exhausted and fell repeatedly, necessitating termination of the experiment after 15 min. Thus while balance and motor coordination appear unaffected by the knockout, stamina was considerably reduced. When evaluated singly in a spontaneous-activity open field test, the KO mice were less inclined to explore the environment and adopted the inquisitive rearing posture less frequently than did the HF control mice. Many of the KO mice walked with splayed rear legs and dragged their posterior on the ground. Shivering was commonly observed. Overall activity of the KO mice in the open field test was much lower than HF controls (Fig. 2, panel F). When placed in an open field as a group, HF control mice immediately separated and began investigating the area. In contrast, the KO mice showed no interest in exploring their environment and remained huddled together, as if seeking warmth and indeed body temperature measurements confirmed that these KO mice suffered from hypothermia (Fig. 2, panel G). KO mice of either sex that were not treated with tamoxifen did not develop any of the phenotypic changes described above.

**Table 1:** Analysis of fecal, plasma and tissue composition.

Source	Parameter	Control	KO
Feces	Lipid, % dry weight	3.2±0.2 (4)	3.2±0.4 (4)
	Protein, % dry weight	9.7±1.8 (4)	10.2±1.4 (4)
Plasma	Sodium, mM	146.5±4.2 (6)	155±9.1 (6)
	Potasium, mM	7.43±2.10 (6)	6.25±1.42 (6)
	Chloride, mM	107.2±4.2 (6)	121±6.7 (6)**
	Calcium, mg.dL^−1^	10.3±1.15 (6)	9.93±1.04 (6)
	Phosphorus, mg.dL^−1^	9.85±2.19 (6)	11.6±4.0 (6)
	Total Protein, g.dL^−1^	5.75±0.72 (6)	5.1±1.08 (6)
	Albumin, g.dL^−1^	3.40±0.39 (6)	3.30±0.59 (6)
	Glucose, mg.dL^−1^	187±45 (11)	278±107 (20)**
	Lactate, mM	13.3±6.1 (14)	20.0±7.8 (19)*
	ß-hydroxybutyrate, mM	0.15±0.07 (8)	0.34±0.14 (13)**
	Glycine, µM	243±88 (3)	465±148 (3)
	Proline, µM	129±43 (3)	414±195 (3)
	Urea Nitrogen, mg.dL^−1^	25.3±3.8 (6)	35.1±12.9 (6)
	Creatinine, mg.dL^−1^	0.112±0.032 (6)	0.10±0.037 (6)
	Total Bilirubin mg.dL^−1^	0.115±0.018 (6)	0.107±0.040 (7)§
	Alkaline Phosphatase U/L	63.4±14.6 (6)	91.6±64.0 (6)
	Alanine Aminotransferase U/L	121.0±62.5 (6)	54.7±13.9 (6)*
	Aspartate Aminotransferase U/L	225±195 (6)	273±207 (6)
	Amylase, U/L	3530±1450 (6)	3436±590 (6)
	Cholesterol, mg.dL^−1^	165±64 (6)	115±28 (6)
	Triglyceride, mg.dL^−1^	117±66 (6)	68.6±57.1 (6)
Liver	Glycogen, mg.g^−1^	26.2±8.2 (8)	21.0±8.3 (9)
Skeletal Muscle	Glycogen, mg.g^−1^	1.08±0.34 (6)	1.68±0.40 (5)*
	Lactate, µmol.g^−1^	0.40±0.2 (5)	6.6±0.4 (7)**

Values for plasma glucose and lactate and for fecal compositions were pooled from males and females; there was no significant difference in these values between the sexes. All other values were obtained from male mice.

§ Two of the seven KO mice were anemic with prolapsed rectums and had bilirubin levels of 0.073±0.056. The five non-anemic KO mice had bilirubin levels of 0.12±0.027. Statistically significant differences are noted:

* p<0.05,

** <0.005.

A survey of the literature on the use of the tamoxifen-inducible B6.Cg-Tg(cre/Esr1)5Amc/J strain in the absence of a floxed target-gene did not uncover any reports of long-term phenotypic changes resembling those identified in our study. Thus, in order to minimize the number of animals required for the study, we considered it unnecessary to include mice carrying the *Cre* gene but lacking the floxed *Mcat* gene. It has been reported that tamoxifen administration to mice carrying the inducible *Cre* can lead to transient cardiomyopathy and changes in energy metabolism, even in mice lacking a floxed transgene [29]. However, this condition is fully reversible within one month of tamoxifen withdrawal. Since the phenotypic changes observed in our KO mice were observed only after tamoxifen-treatment, were not evident until several months after cessation of tamoxifen treatment and were persistent for the lifespan of the animals, we can confidently attribute the changes specifically to the disruption of the *Mcat* gene.

### Necropsy and histology findings: KO mice loose white adipose fat

The most conspicuous feature of the phenotype revealed by gross examination at necropsy was the almost complete absence of white adipose tissue by 7 months post-tamoxifen treatment. Indeed mature visceral and subcutaneous white adipose could only be identified on histological examination. In contrast, there was no significant loss of brown adipose tissue (Fig. S2), which plays an important role in thermogenesis rather than energy storage and originates from a different cell lineage than does white adipose [30]. With the exception of the spleens of a sub-population of KO mice, which were considerably larger than those of control HF mice, there was no statistically significant difference in absolute weights of other organs between KO and HF mice (Fig. S2).

**Figure 3:**
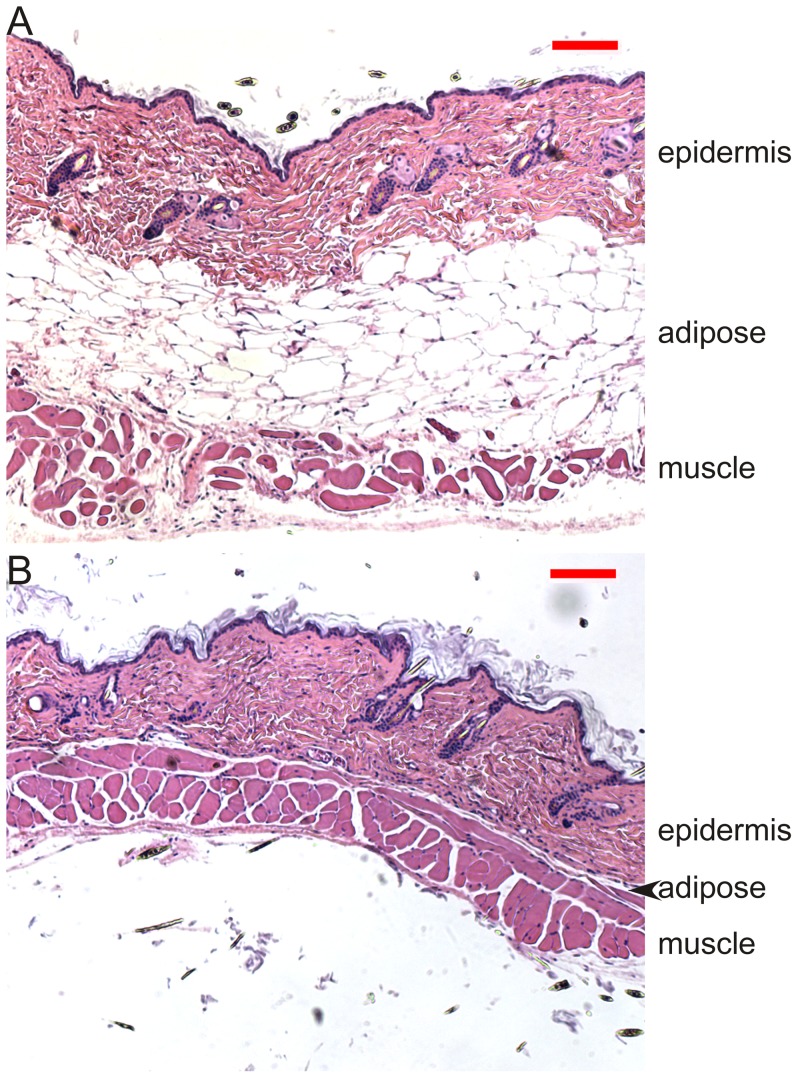
Skin Histology. Dorsal skin sections from HF control (A) and KO (B) mice were fixed, paraffin embedded, sectioned at 10 µm and stained with Hematoxylin and Eosin; bar, 100 µm.

Histological examination of skin removed from KO mice by biopsy revealed a severely depleted subcutaneous adipose layer as well as hyperkeratinization (Fig. 3). It is well recognized that cycling of the hair follicle through phases of intense mitotic activity (anagen), regression (catagen) and dormancy (telogen) is accompanied by expansion and contraction of the subcutaneous adipose layer. New evidence indicates that adipose precursor cells within the dermal fat layer are directly responsible for driving activation of follicular stem cells and induction of the anagen phase, probably by the expression and secretion of platelet-derived growth factor [31]. Other studies have uncovered a correlation between atrophy of subcutaneous adipose tissue and hair loss [32], [33]. Although we cannot rule out the possibility that specific biochemical defects in hair follicles and/or sebaceous glands contribute to hair loss, it seems plausible that in our KO mouse model, the loss of subcutaneous fat plays a role in the development of alopecia. Clearly, loss of the insulation normally afforded by both hair and subcutaneous adipose must place an additional burden on the energy requirements of the KO mice and contribute to the development of hypothermia.

We did not notice any unusual morphologic changes in muscle, bone, or connective tissue in the spinal column, or hind limbs that might account for the observed kyphosis, poor posture or abnormal gait of the KO animals. Nevertheless, it remains possible that subtle changes may have occurred that were not readily apparent in routine sections stained with Hematoxylin and Eosin.

### A subpopulation of knockout mice develop rectal prolapse and anemia

**Figure 4:**
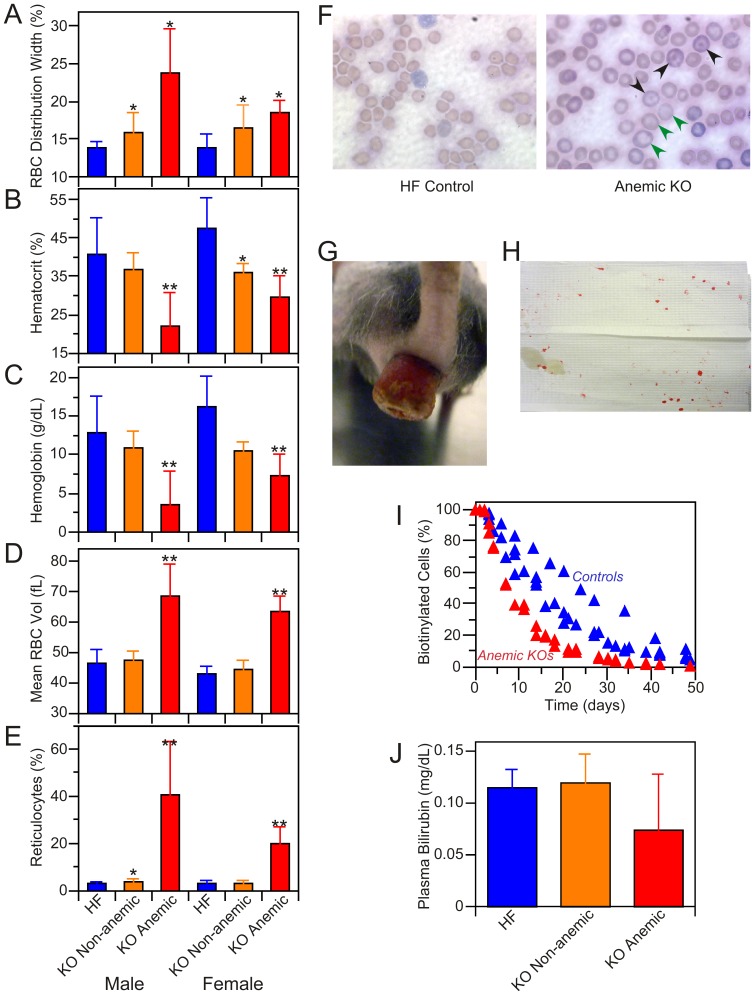
Characterization of anemic KO mice. Approximately 20% of the KO mice were found to be anemic. A–E: blood cell analysis. A: Red blood cell distribution width. Anemic KO mice (5 males and 5 females) were distinguished from non-anemic KO (20 males and 11 females) and HF control (10 males, 9 females) on the basis of lower hematocrit (panel B), lower hemoglobin level (panel C), enlarged red cells (panel D) and elevated reticulocyte levels (panel E). **Significantly different (p<0.05) from non-anemic KO and HF control mice; *significantly different from HF controls. None of the parameters were significantly different between male and female KOs. No major differences were seen in other blood cell components. The complete blood count analysis is presented as Table S1. F: Wright's-stained blood smears from anemic KO and HF control male mice. Green arrows indicate enlarged erythrocytes, black arrows codocytes. G: Rectal prolapse in KO female, 9 months post-induction; 9 of the 10 anemic mice exhibited rectal prolapse. H. Blood spots on cage floor blot (∼9″×6″) obtained by 5 min exposure to 3 anemic mice with rectal prolapse; no blood spots were observed on blots from cages housing non-anemic KO or HF mice. I: Red cell turnover study performed on 2 anemic KOs and 4 HF control female mice. The two anemic mice had reticulocyte levels of 13.4 and 20.2%. J: Plasma bilirubin levels in HF (n = 6), non-anemic KO (n = 5) and anemic KO (n = 2) mice; differences between groups were not statistically significant.

Twenty percent of male and 30% of female KO mice were anemic, exhibiting elevated red blood cell distribution widths, low hemoglobin levels, low red cell counts, increased mean corpuscular volume and elevated reticulocyte levels (Fig. 4, A–E). Reticulocytes derived from anemic KO mice were larger than were those from non-anemic KO and HF control mice as is typically found when intense stimulation of erythropoiesis results in the premature release of ‘stress’ reticulocytes into the circulation [34]. In contrast, only minor differences were observed in white cell levels between KO and HF control mice (Table S1). Analysis of peripheral blood smears from anemic KO mice confirmed the presence of enlarged red blood cells and also revealed the presence of codocytes. These abnormal erythrocytes typically result from a decreased content of hemoglobin that manifests as a pallid ring flanking central and peripheral hemoglobinized zones (Fig. 4, panel F). The anemic KO mice corresponded to the subpopulation exhibiting enlarged spleens (Fig. S2) and showed evidence of lymphoid atrophy and prominent extramedullary hematopoiesis. Predominantly myeloid hematopoiesis was noted in the bone marrow from anemic KO mice. Siderocytes, which contain iron granules not associated with hemoglobin and can be indicative of abnormal erythropoiesis, were absent from spleen and bone marrow in these mice.

Nine of the ten anemic mice also developed severe rectal prolapse, a condition not observed in either the non-anemic KO or HF control animals (Fig. 4, panel G). Histological examination of the prolapse revealed signs of neutrophil invasion and inflammation (details not shown). Replacement of bedding with filter paper revealed blood spots originating from the prolapsed anemic KO mice but not from either the non-anemic KO or HF control animals (Fig. 4, panel H). We were unable to detect blood in feces from prolapsed anemic KO mice indicating that the blood spots originated from the inverted rectum. Biotin labeling experiments revealed that the lifespan of red cells was reduced in the anemic KO mice (Fig. 4, panel I). Based on the calculated half-lives of 7.5 d and 18 d for red cells in the anemic KO and HF control mice, respectively, and assuming that blood volume amounts to ∼7% of body mass, we calculated that the loss of red cells in the prolapsed, anemic mice exceeded that expected from normal turnover by ∼60 µL per day. There was no evidence of erythrophagocytosis in any tissue that might indicate increased removal of defective or senescent erythrocytes; neither were plasma bilirubin levels elevated in the anemic KO mice (Fig. 4, panel J), as would be expected for a hemolytic anemia. Thus the anemia likely results primarily from chronic blood loss. Indeed mice subjected to repeated bleeding develop a macrocytic anemia similar to that described here [35]. Nevertheless, since red blood cell distribution width, a parameter that normally rises with age and is a strong predictor of mortality in humans [36], is greater in both anemic and non-anemic KO mice than in HF mice, we cannot unequivocally rule out the possibility that a defect in erythropoiesis also contributes to the anemia.

### Knockout mice have elevated plasma lactate and ketone bodies

Blood chemistry analysis revealed that none of the markers for liver function (alkaline phosphatase, alanine amino transferase, aspartate amino transferase and bilirubin), kidney function (blood urea nitrogen, creatinine and electrolytes) or pancreatic function (amylase) were significantly altered in the KO mice (Table 1). Although some differences were observed in blood lipid content between KO and HF mice, they were not statistically significant and all values fell within the range typically reported in the literature for normal mice. Nevertheless, several blood chemistry parameters were significantly elevated in KO animals, namely lactate, ß-hydroxybutyrate and glucose, although the range of glucose concentrations was far broader in the KO group (53–656 mg/dL) than in the HF group (121–284 mg/dL). Lactate levels were also significantly elevated in skeletal muscle from KO mice. There were no significant differences in any of the blood chemistry parameters between the sexes. In contrast to the dramatic loss of fat reserves, glycogen stores were not depleted in the KO mice; in fact the skeletal muscle glycogen levels were higher in the KO compared to HF animals. These observations indicated that the flux of glucose and gluconeogenic precursors into glycogen was not impaired in KO mice.

### Compromised fatty acid synthesis reduces availability of precursors for lipoylation of key mitochondrial enzymes

The failure of KO mice to thrive, despite the ingestion of more food than control animals, their loss of white adipose fat, the development of hypothermia and elevated plasma lactate and ß-hydroxybutyrate suggested that their metabolic efficiency was compromised by the genetic defect induced in *Mcat*. Given the earlier finding that the mitochondrial pathway for fatty acid synthesis can supply the octanoyl precursor required for posttranslational lipoylation of key mitochondrial proteins involved in the citric acid cycle [9], [10], we sought evidence as to whether the energy disequilibrium might be attributable to a defect in the mitochondrial protein lipoylation pathway.

**Figure 5:**
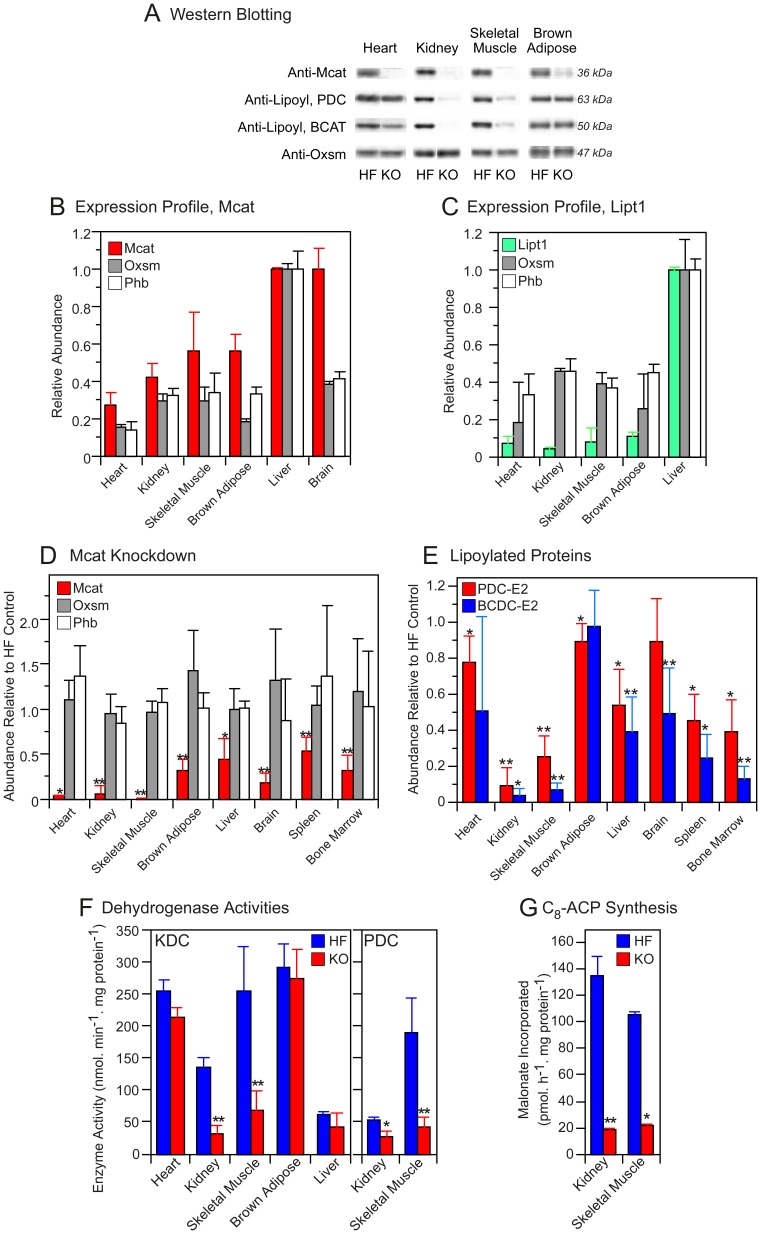
Content and enzyme activity of mitochondrial proteins. A: typical Western blots of mitochondrial proteins illustrating knockdown of Mcat (row 1), lipoylation status of the E2 subunits of PDC and BCDC (rows 2 and 3, respectively), and the content of Oxsm, as a control (row 4). B and C: Expression profiles of Mcat (B) and LipT (C) compared to controls, Oxsm and prohibitin, in mitochondria of HF control mice. The abundance of the proteins is expressed relative to that of liver, which is assigned value of 1.0 (B, n = 2–6; C, n = 1–4). D: Abundance of Mcat and control proteins in mitochondria from KO mice (n = 3–7) relative to the content in HF mice (n = 2–6). E: Lipoylation status of the E2 subunits of PDC and BCDC in KO mice (n = 3–7) relative to HF controls (n = 2–6); mean assigned value of 1.0. F: Activities of KDC and PDC in mitochondria isolated from HF (n, 3–8) and KO mice (n, 4–10). G: Overall activity of the mitochondrial fatty acid synthesis pathway, reported as production of octanoyl-ACP from malonate (n = 2, HF and KO).

Using quantitative Western blotting, we first established that Mcat, as well as ß-ketoacyl synthase (Oxsm), another enzyme required for the mitochondrial fatty acid biosynthetic pathway, is expressed broadly in tissues of HF control mice (Fig. 5, panel A and B). In contrast, we found that expression of LipT1, the lipoyl transferase required for the alternative lipoylation pathway that utilizes exogenous free lipoate, was limited mainly to liver (Fig. 5, panel C). Compared to HF controls, KO mice that had been treated with tamoxifen displayed significantly reduced abundance of Mcat in all tissues examined (Fig. 5, panel D) but no change in the relative abundance of LipT in any tissue (data not shown). The most effective Mcat knockdown was observed in skeletal muscle, where the residual levels were less than 1% of those found in HF controls, and in heart and kidney. The relative abundance of Oxsm, a mitochondrial matrix protein, and prohibitin, a mitochondrial inner-membrane protein, was unaffected by the knockout (Fig. 4 panel D), neither was the yield of mitochondria from various tissues significantly different in the HF and KO mice (details not shown). In addition, the fatty acid composition of kidney mitochondria from KO mice, was found to be very similar to that from HF mice (Fig. S3), despite the effective knockdown of Mcat. The profile of mitochondrial lipid classes was also quite similar, with the exception that cardiolipin levels appear to be elevated in the KO animals (Fig. S3).

The lipoylation status of components of the mitochondrial lipoamide subproteome subproteome was assessed using antibodies directed toward the lipoyl moiety [10]. The lipoylation status of the E2 subunits of PDC and BCDC was affected to variable extents in different tissues; skeletal muscle and kidney suffered the greatest decrease in the abundance of these lipoylated proteins (Fig. 5, panel E). The lipoylation state of the H protein of the glycine cleavage system was also reduced in tissues from the KO mice but the levels were too low to be quantitated reliably by Western analysis (details not shown). Plasma glycine concentration, which is elevated in glycine cleavage system deficiency in humans [37], was also marginally higher in the KO compared to HF mice (Table 1; T-test p value 0.1).

Since the lipoylated E2 subunit of KDC is not recognized by the anti-lipoyl antibodies [10], we assayed the activity of this enzyme directly and found that it too was reduced most effectively by the knockout in skeletal muscle and kidney (Fig. 5. Panel F). Measurement of PDC enzyme activity in skeletal muscle of the KO mice (22.6% of the HF values) showed a good correlation with the extent of the lipoylation knockdown (24.9% of the HF values). Finally we assessed the effect of the Mcat knockdown on the fatty acid biosynthetic capacity of mitochondria, using [2-^14^C] malonate as substrate. The ability of mitochondria to synthesize octanoyl-ACP, the major product of the pathway, was reduced by more that 80% in organelles obtained from kidney and skeletal muscle of KO mice, compared to HF controls (Fig. 5, panel G), consistent with the observation that these tissues have a severe knockdown of Mcat and exhibit a marked lipoylation defect.

### Mitochondrial respiration is compromised by defects in both protein lipoylation and respiratory complexes

**Figure 6:**
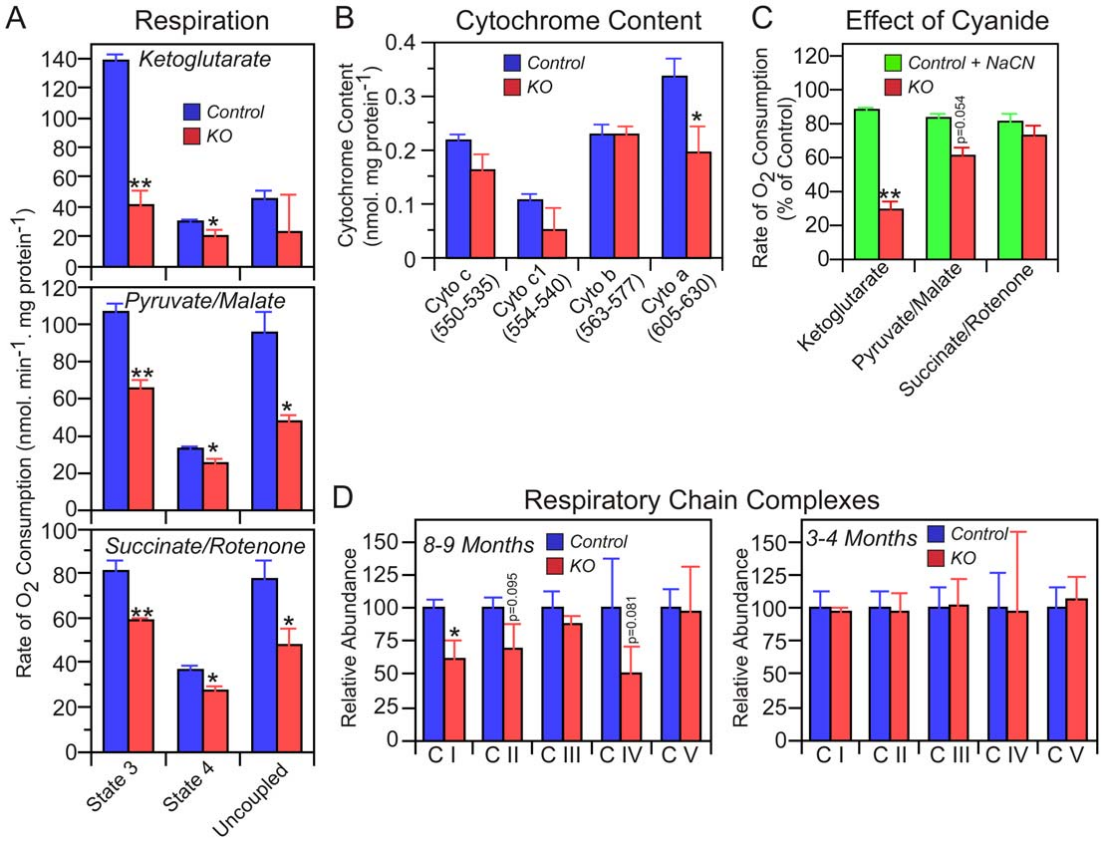
Respiratory properties of skeletal muscle mitochondria. A: Oxygen consumption by mitochondria isolated from mice 8–9 months following treatment with tamoxifen. Values are means ± SEM (n = 4). “State 3” indicates respiration rates during ATP synthesis, “State 4” is in the presence oligomycin to inhibit ATP synthesis, and “uncoupled” is in the presence of carbonyl cyanide p-[trifluoromethoxy]phenylhydrazone to measure maximal respiration rates. The uncoupled rate with α-ketoglutarate is low because the enzyme was depleted of ADP, its allosteric activator; additional data (not shown) demonstrated that addition of ADP increased the uncoupled rate. B: Cytochrome content: values are means ± SEM (n = 5). C: Respiration of mitochondria treated with NaCN. NaCN (15 µM) was added to control mitochondria to mimic the 40% reduction in cytochrome oxidase observed in mitochondria from KO compared to control mice. Respiration, measured in the presence of ketoglutarate, pyruvate+malate, and succinate+rotenone, was compared with that of untreated mitochondria from KO mice. D: Abundance of subunits of the five respiratory chain complexes in mitochondria from KO (n = 3) relative to HF control (n = 4) mice. Western blotting was used to detect subunits of complexes I (NDUFB8), II (30 kDa subunit), III (core protein 2), IV (subunit 1) and V (alpha subunit), employing primary antibodies obtained from Mitosciences. The antigen targets represent labile subunits that are degraded when not assembled into the appropriate complex and thus are representative of the abundance of the fully assembled complexes. Mitochondria were isolated from two groups of mice 8–9 months and 3–4 months following treatment with tamoxifen. Statistical significance in unpaired *t*-tests is indicated by *p>0.05 or **p<0.005; for p values of marginal significance actual values are shown.

Defective lipoylation of PDC and KDC would be expected to limit the ability of mitochondria to respire using these substrates, and indeed respiration on α-ketoglutarate and pyruvate/malate was decreased significantly in mitochondria isolated from skeletal muscle of KO compared to HF mice 200–240 days following tamoxifen treatment (Fig. 6, panel A). Unexpectedly, succinate-driven respiration was also decreased in the KO animals, suggesting the presence of an additional deficit in these mitochondria. Since succinate is metabolized by a non-lipoylated enzyme, succinate dehydrogenase, we asked whether the deficit might result from additional defects in the electron transport chain. Spectral analysis indicated that there was ∼40% reduction in the amount of cytochrome *a*, the redox center of complex IV (cytochrome c oxidase), in skeletal muscle mitochondria from KO mice (Fig. 6, panel B). To assess the effect of this decrease on complex IV activity, we determined the cyanide concentration needed to inhibit complex IV in order to simulate the degree of cytochrome a depletion observed in the KO. This cyanide concentration was then used to quantify the effect of cytochrome a depletion on state 3 respiration employing ketoglutarate, pyruvate/malate, and succinate/rotenone as substrates. The results (Fig. 6, panel C) revealed that the degree of inhibition of respiration driven by succinate/rotenone was not significantly different in mitochondria from KO mice and control mice that had been depleted of cytochrome a. Thus the cytochrome a depletion evident in mitochondria from KO mice could account for the decreased respiration with succinate. In contrast, the decreased respiration rate in mitochondria from KO mice observed with either ketoglutarate or pyruvate/malate as substrates could not be accounted for by the simulated cytochrome a depletion. Thus, these effects can be attributed to defective lipoylation of KDC and PDC. Western analysis of representative subunits from each of the five respiratory complex also revealed lower levels of complexes I, II and IV, although with marginal statistical significance in some cases (Fig. 6, panel D, left). Collectively, these findings indicate that defects in respiratory chain complexes could contribute to the lower respiratory function of mitochondria in skeletal muscle from the KO mice. Knockdown of the mitochondrial pathway for fatty acid synthesis in yeast also has been observed to result in defective assembly of respiratory complexes but the mechanism is unknown [38], [39], [40]. So, how significant are these secondary effects on the electron transport chain in skeletal muscle mitochondria likely to be in the development of the overall phenotype? Firstly, the deficit in respiratory chain complexes is not universal in tissues of the KO mice since no significant changes were observed in kidney mitochondria (Fig. S4). Secondly, within 100 days of tamoxifen treatment, maximum knockdown of the targeted Mcat is achieved and the lipoylation defect is well established (details not shown). At this stage, the mice are significantly smaller than the HF controls (Fig. 2 panels A and B) and already have visible hair loss (Fig. 1 panel F). However no changes in the components of the electron transport chain were apparent by Western blot at this stage (Fig. 6, panel D, right). Thus, the delayed appearance of the defects in the electron transport chain observed in skeletal muscle may exacerbate the severity of the mature knockout phenotype but is unlikely to play a role in the initial development of the phenotype.

### Heart-specific knockout of Mcat is benign

We also engineered a second transgenic mouse model, replacing mice of the B6.Cg-Tg(cre/Esr1)5Amc/J strain with B6129-Tg(Myh6-cre/Esr1)1Jmk mice, in order to produce an Mcat knockout restricted to heart muscle. These mice exhibited a similar level of Mcat knockdown and compromised protein lipoylation in heart as described for the ubiquitous Mcat KO mice, except that no Mcat knockdown was observed in other tissues. These mice had normal appearance and were indistinguishable from the HF mice, according to the general parameters described in Figs. 1 and 2, and were not subjected to any additional testing.

## Conclusions

The most striking characteristic of the Mcat-knockout phenotype is the energy disequilibrium. Affected mice consume more food than control animals, yet they fail to gain weight and are less physically active, they have reduced muscle strength and stamina, they eventually lose all of their white adipose tissue, develop alopecia and kyphosis, loose weight and have a shortened lifespan. Despite their retention of normal brown adipose tissue depots and their shivering behavior, the KO mice are unable to maintain normal body temperature. Biochemical analyses strongly suggest that the primary factor responsible for development of this phenotype is a diminished capacity of tissues such as skeletal muscle to process the products of glycolysis and fatty acid oxidation through the citric acid cycle. Thus the KO animals have elevated levels of skeletal muscle lactate, blood lactate and ketone bodies. The mitochondrial pathway for fatty acid synthesis appears dedicated to the production of octanoyl moieties, the lipoyl precursor, and has limited ability to form longer-chain acyl moieties [3], [9]. Consistent with this concept, failure of the pathway, though knockout of the *Mcat* gene, manifests primarily as a lipoylation defect, rather than as altered mitochondrial fatty acid composition. The amounts of the catalytically-active lipoylated forms of all four components of the mitochondrial lipoamide subproteome, the E2 subunits of the PDC, KDC and BCDC dehydrogenases, and the H-protein component of the glycine cleavage system (GCSH) are reduced in tissues of KO mice, most notably in skeletal muscle and kidney. These results indicate that the de novo pathway for generation of lipoyl moieties is vital for normal energy metabolism.

Prior to the discovery of this pathway it was generally assumed that in mammals lipoyl moieties employed in the posttranslational modification of mitochondrial proteins were derived from free lipoate, which is first activated to lipoyl-AMP and then transferred to the lysine acceptor residue at the lipoylation site. The two enzymes required for this process, a lipoate activating enzyme and lipoyl transferase (Lipt1) have been characterized from bovine liver mitochondria [11], [12]. In contrast, the *de novo* pathway operates through different intermediates: an octanoyl moiety is translocated from thioester linkage on the ACP to the lysine residue on the acceptor protein, where the two sulfur atoms are subsequently introduced by lipoic acid synthase (Lias). Although this enzyme has yet to be characterized in mammals, a constitutive knockout of the *Lias* gene in mice was found to be lethal in early embryonic development [41]. Furthemore, downregulation of the pathway for mitochondrial fatty acid synthesis in human cell culture compromised the protein lipoylation pathway and resulted in cell death [10]. Lipoate supplementation of the diet fed to the pregnant mother did not rescue the knockout mouse embryos neither did lipoate supplementation of the culture medium improve survival of cultured HEK293 cells [10], [41]. These findings, together with the observation that the Mcat-deficient mice exhibit a lipoylation defect, raise the question as to why, with two independent pathways available for protein lipoylation, down-regulation of the *de novo* pathway is not compensated for by the utilization of free lipoate. The answer appears to be that, whereas the enzymes required for the *de novo* pathway are expressed broadly in many different tissues, the lipoyl transferase required for the free lipoate pathway is expressed predominantly in the liver and is not inducible in either liver or extrahepatic tissues when Mcat is compromised. Although we did not attempt rescue of the Mcat-deficient phenotype by lipoate supplementation, in view of the earlier failure of this approach in other model systems it seems likely that restricted expression of the Lipt1 limits the ability of the lipoate scavenging pathway to service universally the components of the mitochondrial lipoamide subproteome.

As is the case with many inducible gene knockout animal models, the tamoxifen-induced ablation of the *Mcat* gene is incomplete so that the residual enzyme expression ranges from 0.2% in skeletal muscle to 53% in spleen. Additionally, as discussed above, in some tissues the Mcat defect results in only mildly compromised protein lipoylation so that the phenotypic changes observed in the *Mcat* knockout model are milder than would be anticipated for a general, constitutive knockout. Retention of some protein lipoylation capacity in tissues such as liver also allows for further metabolism of lactate and ketone bodies released from tissues that are compromised by a severe lipoylation defect. Consequently, the effects of the Mcat knockout are less grave than total knockouts of components of the mitochondrial lipoamide subproteome, which can lead to severe neurological and cardiac defects [42], [43], [44], [45]. Mutations in components of the mitochondrial pathway for fatty acid synthesis that produce a lethal null phenotype, as observed in the knockout of mouse Lias, the terminal enzyme in the *de novo* pathway for protein lipoylation, are unlikely to manifest as disease in the human population. Therefore, one can argue that the hypomorphic phenotype observed with the inducible *Mcat* knockout represents a more useful model for predicting the consequences of a non-lethal defect in the pathway in the human population. In the *Mcat* KO model, the degree of Mcat knockdown and compromised protein lipoylation is particularly severe in skeletal muscle. Indeed, muscle weakness and poor posture is a readily recognizable characteristic of the phenotype and may play a role in the development of both kyphosis, through weakening of the paraspinal muscle [46], and rectal prolapse, through weakening of muscles of the anal sphincter and those that hold the rectum in place [47].

Because of the crucial role played by the citric acid cycle in energy metabolism, the primary defect in lipoylation of PDC and KDC resulting from the deficiency in Mcat in some tissues may initiate a cascade of secondary effects that could contribute to the phenotype. In skeletal muscle, where the lipoylation pathway is severely effected, a compromised citric acid cycle could limit the availability of citrate for export into the cytosol where it provides the acetyl-CoA required for de novo fatty acid synthesis via the type I fatty acid synthase. Triglycerides synthesized and stored in muscle cells are now recognized as an important fuel for muscle activity during exercise [48]. Other less predictable secondary effects, such as the observed late-developing depletion of components of the electron transport chain in muscle, may also contribute to the phenotype. Detailed evaluation of these secondary effects resulting from the lipoylation defect in different tissues was beyond the scope of this initial study. A summary highlighting the features of the Mcat-deficient phenotype is presented in Box 1.

Many of the phenotypic characteristics of the *Mcat* knockout mouse model bear striking resemblance to those described in other models of compromised mitochondrial function that have been collectively termed as examples of accelerated aging. For example, mice carrying a proofreading-deficient mitochondrial DNA polymerase exhibit reduced lifespan, weight loss, reduced subcutaneous white adipose, alopecia, kyphosis and an age-dependent, macrocytic anemia with abnormal erythroid maturation [33], [49]. These phenotypic changes result primarily from an accumulation of mutations in the mitochondrial genome that leads to loss of respiratory chain function. Mice deficient in the mitochondrial helicase SupvL1, a component of the mitochondrial RNA degradosome complex, exhibit growth retardation, kyphosis, thickening of the epidermis and atrophy of subcutaneous adipose and muscle mass and early death [50]. In this case the phenotypic changes most likely result from aberrant mitochondrial RNA processing that leads to impaired protein synthesis and ATP production. Recently, a mouse model with compromised mitochondrial superoxide dismutase exclusively in connective tissue has been described that is also characterized by weight loss, osteoporosis and kyphosis, skin abnormalities, muscle loss and reduced lifespan [51]. In this model, the altered phenotype results from oxidative damage in mitochondria and loss of redox balance.

Although it may seem remarkable that random mutations in mitochondrial DNA, defects in the mitochondrial degradosome, deficiency of mitochondrial superoxide dismutase and compromised mitochondrial fatty acid synthesis precipitate a similar set of phenotypic changes, this is one of the hallmarks of mitochondrial diseases: different mutations in either the mitochondrial or nuclear genomes can lead to similar diseases, known as phenocopies. Several phenotypic features of these mouse models of accelerated aging are also associated with aging in humans, particularly osteoporosis, kyphosis, decreased muscle strength, loss of subcutaneous fat resulting in skin wrinkling and alopecia, prolapsed rectum and weight loss, typically after age 60 years. The broad range of mitochondrial defects that can lead to the accelerated aging phenotype serves to illustrate the difficulty in establishing the etiology of human diseases in which defective mitochondrial function is implicated. The present study suggests that current guidelines for the evaluation of patients of all ages with suspected mitochondrial diseases [2] should be broadened to include the possibility of compromised mitochondrial fatty acid synthesis and protein lipoylation.

## Supporting Information

Figure S1 **Engineering of the** ***Mcat*** **knockout gene.** A. Overall strategy. A schematic of the targeting vector (i) showing the locations of the *Frt*-flanked *ß-geo* cassette, *lox* sites, and both the coding (black-filled rectangles) and non-coding (white-filled rectangles) regions of the 4 exons. Targeted ES cells were transfected with a *Flp* recombinase plasmid to excise the gene-trapping cassette creating a “floxed” or conditional knockout allele (ii). Chosen clones were injected into blastocysts, chimeric offspring were identified and bred to homozygosity for the floxed *Mcat* allele. These mice were bred with mice of the inducible-*Cre* strain B6.Cg-Tg(cre/Esr1)5Amc/J. Mice homozygous for the the floxed *Mcat* allele carrying a single copy of the *Cre* gene were identified and treated with tamoxifen to generate a truncated *Mcat* gene (iii). B. Annotated nucleotide sequence of the modified gene. Boxed region: Exon 2. Green text *lox*71 (upstream) and *lox*P (downstream) sites. Blue text: The single remaining *Frt* site after *Flp* recombination. Pink text: Residual exogenous sequence from construct engineering. Yellow highlight: Location of primer pairs used for characterization of the *Mcat* alleles by the PCR. Pairs p5 (CGGTACTGATTACAACCATGAGCGG-CCATAG) and pEx2 (AGCAACACAGTTGTCGATGA-CCTG) or p5 and p3 (CACTCAAGCTGGGT-GCTGAATAGGCTTTGCAA). The *Cre* recombinase gene was detected using the PCR as recommended by Jackson Labs.
(PDF)

Figure S2 **Organ Weights**. Weights of organs from KO mice are expressed relative to those of HF control mice, both as gross weights (blue) and after normalization for body weight (red). Brown adipose is from the interscapular region. Abdominal fat depots in KO mice were too small to be measured. The number of measurements is shown in parenthesis for the HF and KO animals, respectively. The statistical significance, where detected, is indicated by * p<0.05, **p<0.005.
(PDF)

Figure S3 **Analysis of kidney mitochondrial lipids.** Mitochondria were isolated from kidneys, obtained from male KO (22.3 g) and HF (38.6 g) mice 8 months after treatment with tamoxifen, purified by centrifugation on an iodixanol gradient (fraction density ∼1.13 g/mL) and frozen. Thawed mitochondria were washed with 150 mM ammonium bicarbonate. Lipids were extracted with chloroform∶methanol (2∶1) from portions equivalent to 50 µg of mitochondrial protein and analyzed by mass spectrometry, essentially as described earlier [52]. For analysis of total fatty acid composition, mitochondrial lipids were methylated by treating mitochondria with 2.5% H_2_SO_4_ in methanol for 1.5 hour at 85°C. Methyl esters were extracted with hexane and analyzed by gas chromatography. Results are reported as mole % distribution within each lipid class. The total amounts of fatty acid recovered were 133 and 159 ng per mg protein for HF and KO mice, respectively. The total amounts of each lipid class recovered from the KO mouse relative to that from the HF mouse (assigned a value of 1.0) were: cardiolipin, KO 2.0±0.4, HF 1.0±0.04; lysophosphatidylinositol and phosphatidylinositol, KO, 1.3±0.2, HF 1.0±0.01; lyso phosphatidylethanolamine and phosphatidylethanolamine, KO 1.07±0.08, HF 1.0±0.02; phosphatidyl choline, KO 1.3±0.0, HF 1.0±0.05; sphingomyelin, KO 0.65±0.001, HF 1.0±0.1. Trace amounts of phosphatidyl serine and phosphatidic acid were detected that were too low for quantitation. Blue bars correspond to HF control values, red to KO.
(PDF)

Figure S4 **Abundance of subunits of the respiratory chain complexes in kidney mitochondria.** Western blotting was used to detect subunits of complexes I (NDUFB8), II (30 kDa subunit), III (core protein 2), IV (subunit 1) and V (alpha subunit) as described in Figure 6D.
(PDF)

Table S1 **CBC Analysis.** **Significantly different (p<0.05) from non-anemic KOs and HFs; *significantly different from HFs. None of the parameters were significantly different between male and female KOs.
(PDF)
