# Supplementary material for: Compromised Mitochondrial Fatty Acid Synthesis in Transgenic Mice Results in Defective Protein Lipoylation and Energy Disequilibrium
Source: PLoS One. 2012 Oct 15;7(10):e47196. doi: 10.1371/journal.pone.0047196 (PMC3471957; doi:10.1371/journal.pone.0047196)
Supplement: Figure S1 — Engineering of the Mcat knockout gene. A. Overall strategy. A schematic of the targeting vector (i) showing the locations of the Frt-flanked ß-geo cassette, lox sites, and both the coding (black-filled rectangles) and non-coding (white-filled rectangles) regions of the 4 exons. Targeted ES cells were transfected with a Flp recombinase plasmid to excise the gene-trapping cassette creating a “floxed” or conditional knockout allele (ii). Chosen clones were injected into blastocysts, chimeric offspring were identified and bred to homozygosity for the floxed Mcat allele. These mice were bred with mice of the inducible-Cre strain B6.Cg-Tg(cre/Esr1)5Amc/J. Mice homozygous for the the floxed Mcat allele carrying a single copy of the Cre gene were identified and treated with tamoxifen to generate a truncated Mcat gene (iii). B. Annotated nucleotide sequence of the modified gene. Boxed region: Exon 2. Green text lox71 (upstream) and loxP (downstream) sites. Blue text: The single remaining Frt site after Flp recombination. Pink text: Residual exogenous sequence from construct engineering. Yellow highlight: Location of primer pairs used for characterization of the Mcat alleles by the PCR. Pairs p5 (CGGTACTGATTACAACCATGAGCGG-CCATAG) and pEx2 (AGCAACACAGTTGTCGATGA-CCTG) or p5 and p3 (CACTCAAGCTGGGT-GCTGAATAGGCTTTGCAA). The Cre recombinase gene was detected using the PCR as recommended by Jackson Labs. (PDF) [file pone.0047196.s001.pdf]

ATCTCTCTCTCTTCAGCCCTTTGAAAATGTTTTTAAAAGATGAGTTCCTTAAATGAAAGATACTAAGAAATTGATGTTAGGGGCTCAGGGATGGCTC
ATCCATTAAGAGTGTTTACTGCTCATGTAGAAGCCAGTTTCAGGCCCCAAGCAAGACCCTGGCTCATAAAATGCTGGTAACTTGAGATTCAAGGGAT
CCTGTGCCCTCTTCCAGCCTCCACGAGCATGTGCCAGGCTTAGAGATGAACACCTTTAATCTCAACACTCAGGAGGCCGAAGCAGGCAGATCTCTCC
AAATTCCAGGCCAGTTGGGGGTACACAGTGAGACCCTGTCTGAAAAGACAGAGATGGCATGGCAAACATTAGCGCATGTGACTTCACTTGCTGCGGA
TATGTTATACTCCCAGGACAACACATGTTAACTGGTGCTGAGACCCTCTGTTCTACCTCCACAGCCACCTTCTGAGGCAATTGTTAGGAGCAATGGG
AGGCTCAGGCGACAGGAATGAACATGATCCTGACGGAAGGCTCAGGTGACAGGAATAAGCATGGCCCTGTGACACATTTCCTCGGTGCTTCTCTGGG
CCAAGCCCGCACTGAGAGTCTATAGATACCAGCATTGCCTACTTATGTTAGCAGTCATGTGAAATAGGTCTGGGGGGACTAAATCTGCTTCTCTGTG
GCTTGGAAAATCATAGTTTTTTAAGTTTAATTTTACTCTAGCTGCATTGGTGTTTTGTCTGCATTCATGTCTCTGAGGGTGTTGGATCCCCTGGAAT
AGAAACAGCTGTGAGCTGCCACAAGGATGCTGGGAATTGAACTAGGGTTCTCTGGAAGAGCAGCCAGTGCTTGTAACTGATGAACCATTTCTCCAGC
CCCCTAGAAAATCATATTTTTAATGCCTGTACTTTTGTTTTGTTTGGAAGCATGGGAAGGGTGAACTGTAATGTAGCTGTGTTGGCACTGAATTCAA
AACTCTTTAGCTTCAGCCTCCTCCGGTACTGATTACAACCATGAGCGGCCATAGGCAGTGTGCATGTAATTTCTGTATCCTATGTGCCCGAGAAGCC
p5>
ACAGAGGATCAGTGCTCTGGCTCATAGTGTAAGGCGCATAACGATACCACGATATCAACAAGTTTGTACAAAAAAGCAGGCTGGCGCCGGAACCCTT
TCCCACACCACCCTCCACACTTGCCCCAAACACTGCCAACTATGTAGGAGGAAGGGGTTGGGACTAACAGAAGAACCCGTTGTGGGGAAGCTGTTGG
GAGGGTCACTTTATGTTCTTGCCCAAGGTCAGTTGGGTGGCCTGCTTCTGATGAGGTGGTCCCAAGGTCTGGGGTAGAAGGTGAGAGGGACAGGCCA
CCAAGGTCAGCCCCCCCCCCTATCCCATAGGAGCCAGGTCCCTCTCCTGGACAGGAAGACTGAAGGGGAGATGCCAGAGACTCAGTGAAGCCTGGG
GTACCCTATTGGAGTCCTTCAAGGAAACAAACTTGGCCTCACCAGGCCTCAGCCTTGGCTCCTCCTGGGAACTCTACTGCCCTTGGGATCC**TACCGT**
TCGTATAGCATACATTATACGAAGTTATGTGATAGGCCTAGGGAAGTTCCTATTCC**GAAGTTCCTATTCTCTAGAAAGTATAGGAACTTC**GTCGAGA
TATCTAGACCCAGCTTTCTTGTACAAAGTGGTTGATATCTCTATAGTCGCAGTAGGCGGGACTGGAATCGACTTTAGCTGTCTTGCTAAGGAAAGCC
CTCATCAATAAAGGGGAGTGAGTACCAGGCCAATAGAATTGGCTCATGGTGACACTTAATTGCCCTGTCAGTTCCAAGGAGGGGGCACATCCCGGG
ACTGAGCTCGGAGGATTCAGCCTCATGTTGTGCTCTGTCTCCGCAGGTCATCGACAACTGTGTTGCTGCGGCTGGCTTCAGTGTGGGAGAGTTTGCT
<pEx2
GCCTTGGTATTTGCTGGAGCCATGGATTTTTCTGAAGGTACACAAGAAGTGTGACTTGTTCAGGGTGTGTTCTCGCTGGGCTCAGTGGGGACAGGGG
ACTCTGTGGACAGTGGTGCTGGATCCACGCAGGGTTGTCGGGAAGGGAGCCCAGGGATGCAGCTGATACTGGAGCTCAAGGACTGGATTCCACCGAC
CCCCTTTGAATCTCAAGGATCCGAGGTGTGTTTGCTTTCTTCCTGTAATTTCATGAGGTCCGCTTTCCTGGCTGCAACCCTTCACTGTCCTCTGCAT
TGTTTTTTTTAATTACATAAGTGTGTGTGTGTGTGTGTGTGTGTATGCATATGCATGCACAGGCCTCAGTGTGTGTGATGTCAAAGGACAATGTT
TAGAGTTGGTCTATTACTTCTAATCTGTGGATTTCTGGGATTGAACTCAGGTTGTTAGGTTTGGCTACAGGCATCTCTACCCACTGAGCCCTCTTGC
TGGCCCTCCCTTTGCATTTTTGTTTTTTCGAGACAGGGTTTCTCTGATTAGCCCTGGCTGTCCTGGAATTCACTCTGTAGACCAGGCTGGTCTCGAA
CTCAGAAATCTGCCTGCCTTTGCCTCTCAAGTGCTGCGACTAAAGGCATGCGCCACCACGCCCAGCCTCCCTTTAAGCCGTTGATCAACAGATCCTC
TACGCGGCCGCGGTACC**ATAACTTCGTATAGCATACATTATACGAAGTTAT**GAATTCATTCCGGTTCGCTTGCTGTCCACATTCTGTGCCTGTCACA
CCTGGAAGATGTTCAGTACCTGTTATGAAAAAAAAGAGTAAATGAATACTTTGATTATTGCCTAGTGTTTGCAAAGCCTATTCAGCACCCAGCTTGA
GTGCAGGGAACTGAGCTTGGATCCTCTTCAAGAGGAGTTAGTACTCCGAGCTGATGGGCTAGCTCTCCAGCCCCATTTCCTAAGATTTTTTTTTTTT
<p3
AAGCTGGAACTGGGCAAAGTATCATATACTTGTAGCCCTGGCTACTCAGGAGGCTGTGGCAAAAAGATCACTTGTGCTGAGCTTGGGACTTGTGCCC
TTAATTCCAGCACTTGGAGAATGAGCAAAGGCAGGGGGGCTAAGTGAGCTCAAGGCAAGCCTGGTCTACATAGCAAGCCCCAGATCCACCGGGGCTA
CAGAGTGAGACCCTGTCTCAAAAAGGGTGGAGGGAGGGCATTTGAGTTCAAGTCTAGTGTAGGGAACATTCATTCTGGGGGGTGGGGGAGAGGGAGG
GAGGGAGAGGCAGAGATAGACAGGACTCCCCCCCCCATACACACACACACACACACACACACAGAGAATATTACACAAGTTCTTGACTGAATGATTC
ATTGATTTAAAATCTGCTTTATAGTTCCGTCCAGGGAAGGGACTTGAACGCACATTTCTAGGGAGGTGAGACCCAGATGGAAGATAAACCCCATGTG
TAACGACACTGGTCAACAGGGAAGTGAAAGACAGCCCACATGAGCAGCACTTCATACCCCATAGGAGATGAAGGTTGAGAGTATGCAGAAGTCAGCC
CAGG
