# Supplementary figures and images for: Compromised Mitochondrial Fatty Acid Synthesis in Transgenic Mice Results in Defective Protein Lipoylation and Energy Disequilibrium
Source: PLoS One. 2012 Oct 15;7(10):e47196. doi: 10.1371/journal.pone.0047196 (PMC3471957; doi:10.1371/journal.pone.0047196)

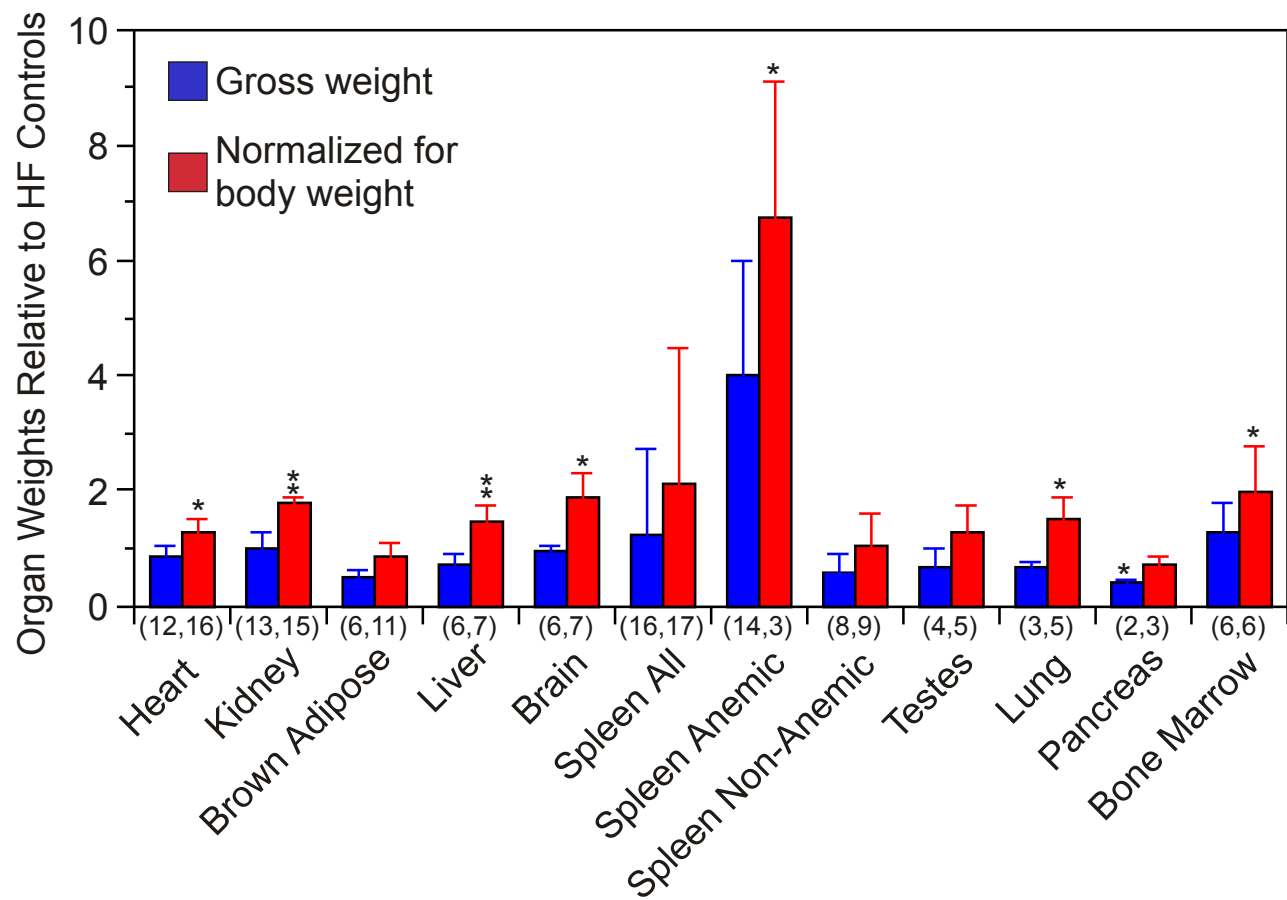

Supplement: Figure S2 — Organ Weights. Weights of organs from KO mice are expressed relative to those of HF control mice, both as gross weights (blue) and after normalization for body weight (red). Brown adipose is from the interscapular region. Abdominal fat depots in KO mice were too small to be measured. The number of measurements is shown in parenthesis for the HF and KO animals, respectively. The statistical significance, where detected, is indicated by * p<0.05, **p<0.005. (PDF) [file pone.0047196.s002.pdf]

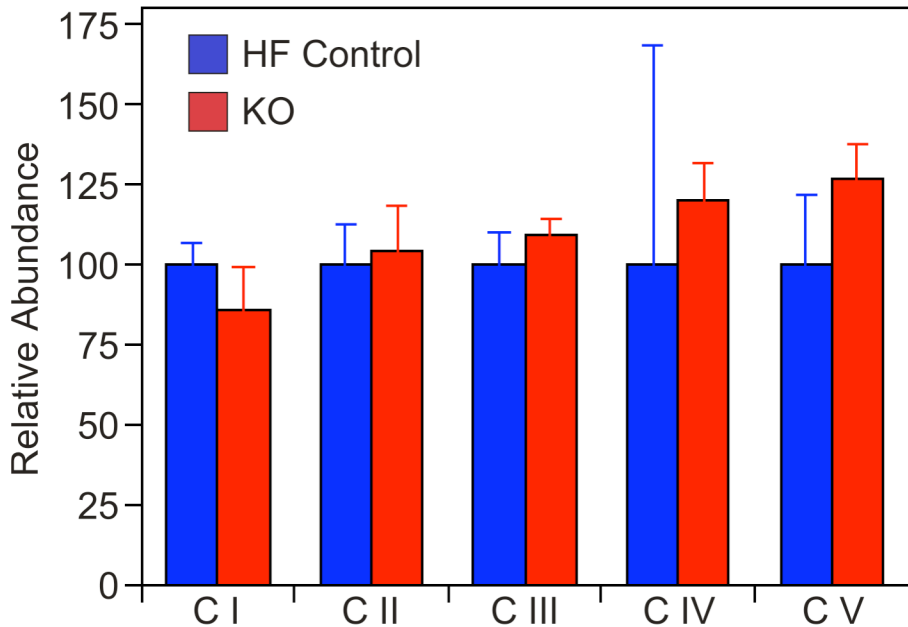

Supplement: Figure S4 — Abundance of subunits of the respiratory chain complexes in kidney mitochondria. Western blotting was used to detect subunits of complexes I (NDUFB8), II (30 kDa subunit), III (core protein 2), IV (subunit 1) and V (alpha subunit) as described in Figure 6D. (PDF) [file pone.0047196.s004.pdf]
