# Supplementary material for: Compromised Mitochondrial Fatty Acid Synthesis in Transgenic Mice Results in Defective Protein Lipoylation and Energy Disequilibrium
Source: PLoS One. 2012 Oct 15;7(10):e47196. doi: 10.1371/journal.pone.0047196 (PMC3471957; doi:10.1371/journal.pone.0047196)

A. Fatty acid composition (%)

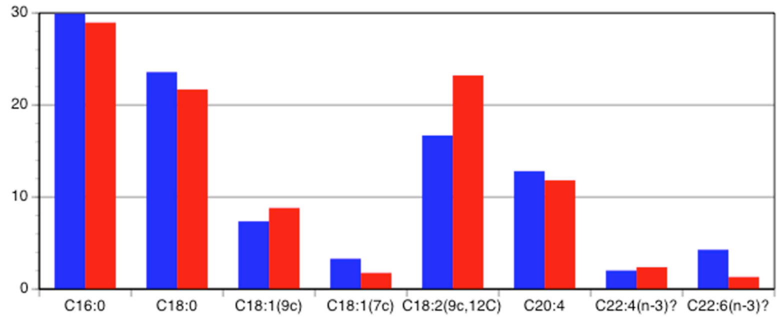

B. Phosphatidyl choline composition (%)

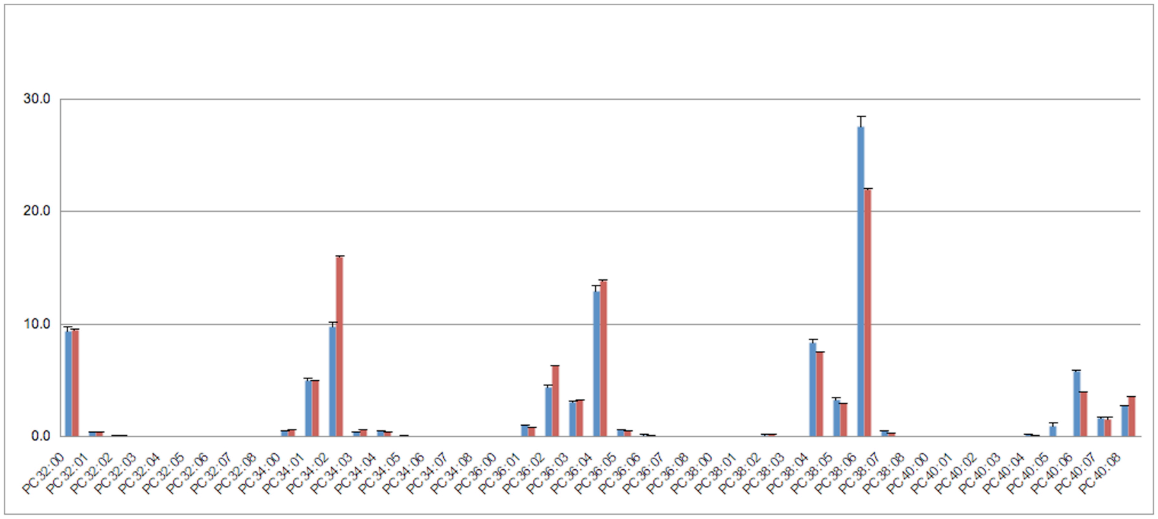

C. Lyso- and phosphatidyl choline composition (%)

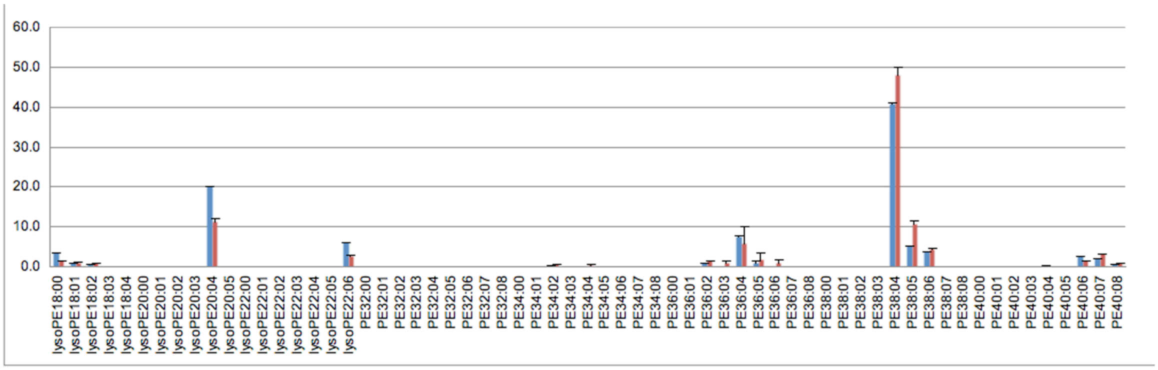

D. Lyso- and phosphatidyl inositol composition (%)

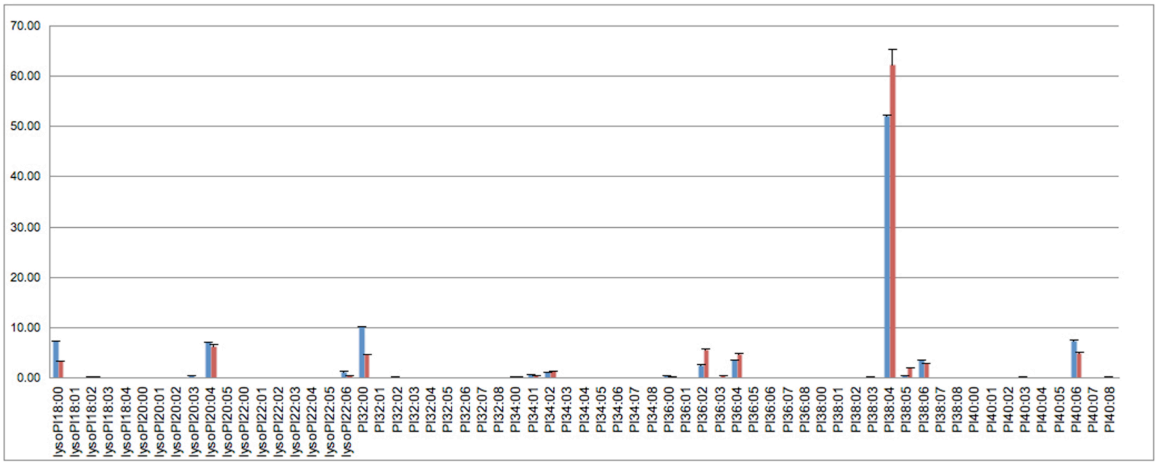

E. Sphingomyelin composition (%)

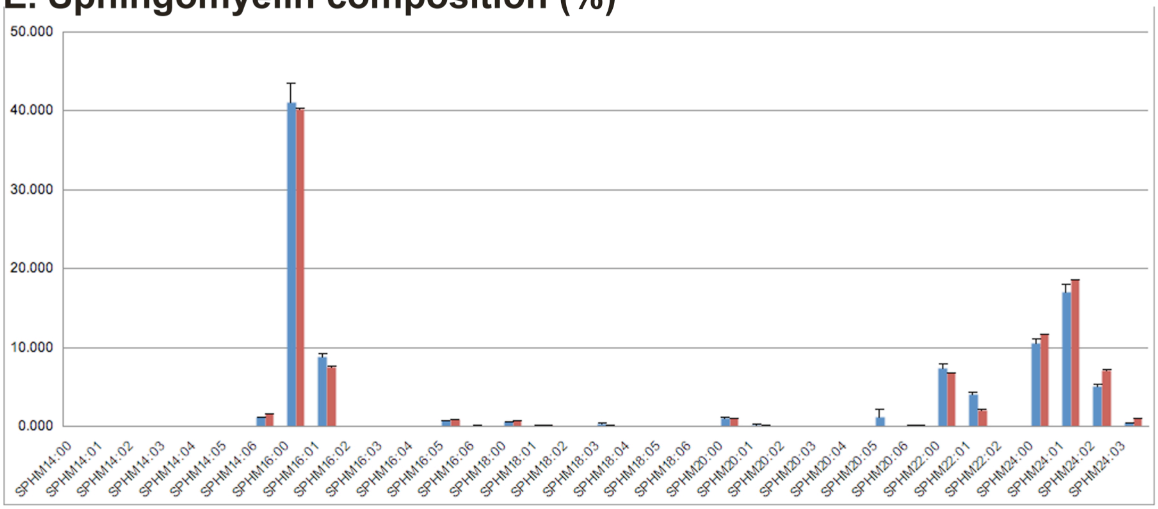

F. Cardiolipin composition (%)

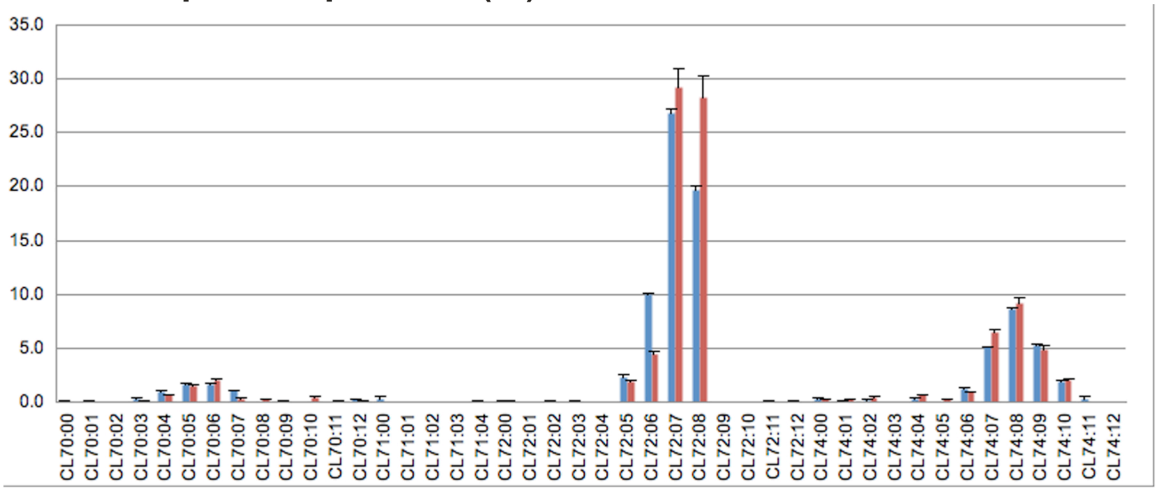

Supplement: Figure S3 — Analysis of kidney mitochondrial lipids. Mitochondria were isolated from kidneys, obtained from male KO (22.3 g) and HF (38.6 g) mice 8 months after treatment with tamoxifen, purified by centrifugation on an iodixanol gradient (fraction density ∼1.13 g/mL) and frozen. Thawed mitochondria were washed with 150 mM ammonium bicarbonate. Lipids were extracted with chloroform∶methanol (2∶1) from portions equivalent to 50 µg of mitochondrial protein and analyzed by mass spectrometry, essentially as described earlier [52]. For analysis of total fatty acid composition, mitochondrial lipids were methylated by treating mitochondria with 2.5% H2SO4 in methanol for 1.5 hour at 85°C. Methyl esters were extracted with hexane and analyzed by gas chromatography. Results are reported as mole % distribution within each lipid class. The total amounts of fatty acid recovered were 133 and 159 ng per mg protein for HF and KO mice, respectively. The total amounts of each lipid class recovered from the KO mouse relative to that from the HF mouse (assigned a value of 1.0) were: cardiolipin, KO 2.0±0.4, HF 1.0±0.04; lysophosphatidylinositol and phosphatidylinositol, KO, 1.3±0.2, HF 1.0±0.01; lyso phosphatidylethanolamine and phosphatidylethanolamine, KO 1.07±0.08, HF 1.0±0.02; phosphatidyl choline, KO 1.3±0.0, HF 1.0±0.05; sphingomyelin, KO 0.65±0.001, HF 1.0±0.1. Trace amounts of phosphatidyl serine and phosphatidic acid were detected that were too low for quantitation. Blue bars correspond to HF control values, red to KO. (PDF) [file pone.0047196.s003.pdf]
