# Supplementary material for: Compromised Mitochondrial Fatty Acid Synthesis in Transgenic Mice Results in Defective Protein Lipoylation and Energy Disequilibrium
Source: PLoS One. 2012 Oct 15;7(10):e47196. doi: 10.1371/journal.pone.0047196 (PMC3471957; doi:10.1371/journal.pone.0047196)
Supplement: Table S1 — CBC Analysis. **Significantly different (p<0.05) from non-anemic KOs and HFs; *significantly different from HFs. None of the parameters were significantly different between male and female KOs. (PDF) [file pone.0047196.s005.pdf]

Table S1. CBC Analysis

Parameter	Males: KO Anemic (n=5)	Males: KO Non-anemic (n=20)	Males: HF (n=10)	Females: KO Anemic (n=5)	Females: KO Non-anemic (n=11)	Females: HF (n=9)
Reticulocytes (%)	40.5±22.9**	3.7±1.2*	2.9±0.7	20.0±6.9**	3.3±1.1	3.1±1.1
Reticulocytes (x 10^{-9} cells/L)	1184±745	287±98	246±71	942±412**	269±88	348±142
Red blood cells (x 10^{-6} cells/µl)	3.42±1.64**	7.7±0.87	8.85±1.83	4.68±0.99**	8.1±0.8*	11.1±1.8
Hemoglobin (g/dL)	5.97±2.57**	12.1±1.7	13.80±2.37	7.28±1.76**	12.2±1.1*	16.6±3.3
Hematocrit (%)	22.2±8.4**	36.7±4.3	40.9±9.2	29.5±5.5**	35.9±2.3*	47.5±7.9
Mean red cell volume (fL)	68.1±10.7**	47.5±2.7	46.2±4.7	63.5±4.7**	44.3±3.1	42.9±2.51
Mean reticulocyte cell vol. (fL)	80.2±14.2**	61.9±3.8	56.7±3.9	73.2±3.7**	58.8±4.4	56.1±1.8
Mean cell hemoglobin (pg)	18.1±3.0	15.72.3	15.6±1.1	15.9±4.0	15.0±1.6	15.0±1.5
Red cell distribution width (%)	23.7±5.9*	15.9±2.5*	13.7±0.9	18.5±1.6*	16.4±3.0*	13.7±2.0
Mean cell hemoglobin (g/dL)	26.9±4.5*	33.0±4.6	34.2±3.6	24.8±5.3*	33.9±2.8	34.8±1.9
Hemoglobin per red cell (pg)	18.1±1.1*	15.8±0.9	15.2±0.68	19.1±0.97**	15.0±1.2	14.9±1.3
White cells (x 10^{-3} cells/µl)	5.2±3.5	5.7±2.2	4.3±2.7	3.9±1.0*	4.5±3.0	6.6±3.1
Neµtrophils (x 10^{-3} cells/µl)	0.89±0.59	0.81±0.57	0.79±1.03	1.07±0.79	0.48±0.28	0.50±0.20
Lymphocytes (x 10^{-3} cells/µl)	3.80±2.98	4.3±2.1	3.14±2.15	2.41±1.32*	3.75±2.79	5.8±2.9
Monocytes (x 10^{-3} cells/µl)	0.28±0.20	0.23±0.14	0.25±0.21	0.20±0.10	0.16±0.13	0.14±0.10
Eosinophils (x 10^{-3} cells/µl)	0.15±0.23	0.14±0.15	0.08±0.08	0.09±0.05	0.09±0.05	0.15±0.08
Basophils (x 10^{-3} cells/µl)	0.01±0.02	0±0	0.01±0.02	0.01±0.02	0.01±0.02	0.01±0.02
Platelets (x 10^{-3} cells/µl)	329±292	501±297	465±389	606±360	438±268	632±294
Mean platelet volume (fL)	9.4±3.0	6.82±1.09	11.8±8.3	6.44±0.67	7.58±1.3	7.99±3.64

**Significantly different ($p<0.05$) from non-anemic KOs and HFs; *significantly different from HFs. None of the parameters were significantly different between male and female KOs.
